# Consequences of the COVID-19 Pandemic on Children's Mental Health: A Meta-Analysis

**DOI:** 10.3389/fpsyt.2021.691659

**Published:** 2021-12-01

**Authors:** Eve-Line Bussières, Catherine Malboeuf-Hurtubise, A. Meilleur, Trinity Mastine, Elodie Hérault, Nicholas Chadi, Marjorie Montreuil, Mélissa Généreux, Chantal Camden, PRISME-COVID Team, Chantal Camden

**Affiliations:** ^1^Department of Psychology, Université du Québec à Trois-Rivières, Trois-Rivières, QC, Canada; ^2^Department of Psychology, Bishop's University, Sherbrooke, QC, Canada; ^3^Department of Psychology, Université du Québec à Montréal, Montreal, QC, Canada; ^4^Centre de Recherche du Centre Hospitalier Universitaire de Sherbrooke, Sherbrooke, QC, Canada; ^5^Division of Adolescent Medicine, Department of Pediatrics, Sainte-Justine University Hospital Centre, Université de Montréal, Montreal, QC, Canada; ^6^Ingram School of Nursing, McGill University, Montreal, QC, Canada; ^7^Department of Psychology, University of Sherbrooke, Sherbrooke, QC, Canada

**Keywords:** COVID-19, children, mental health, meta-analysis, impact

## Abstract

**Background:** The COVID-19 pandemic has exacerbated mental health problems in many individuals, including children. Children with pre-existing socio-demographic or developmental risk factors may be particularly vulnerable to the negative effects of the pandemic and associated public health preventive measures.

**Objective:** This systematic review and meta-analysis explored the impacts of the COVID-19 pandemic on the mental health of children aged 5–13 years-old, while highlighting the specific difficulties experienced by children with neurodevelopmental issues or chronic health conditions.

**Methods:** A systematic search of the published literature was conducted in Medline, ERIC, PsycINFO, and Google Scholar, followed by a quantitative meta-analysis of the eligible studies.

**Results:** Out of the 985 articles identified, 28 empirical studies with prospective or retrospective longitudinal data were included in the quantitative synthesis. COVID-19 lockdown measures were associated with negative general mental health outcomes among children (*g* = 0.28, *p* < 0.001, and *k* = 21), but of small magnitude. Sleep habits were also changed during the pandemic, as sleep duration significantly increased in children (*g* = 0.32; *p* = 0.004, and *k* = 9). Moreover, results did not differ between children from the general population and those from clinical populations such as children with epilepsy, oncology, neurodevelopmental disorders, or obesity. Effect sizes were larger in European vs. Asian countries.

**Conclusions:** Studies included in this review suggest that children's mental health was generally negatively impacted during the COVID-19 pandemic. More research is needed to understand the long-term effects of the COVID-19 pandemic on children's mental health and the influence of specific risks factors as they evolve over time.

## Background

In December 2019, a highly infectious strain of the coronavirus (COVID-19) emerged in China and spread globally within a few months. This led the World Health Organization to declare a global pandemic status (1). In early 2020, several countries implemented lockdown measures, leading to extended school closures, and home lockdown for children and their families. Though lockdown measures were gradually lifted, and some schools were allowed to reopen, children's regular routines were disrupted with the addition of new rules, such as wearing a mask in class or making a transition to online or hybrid schooling (instead of in-person learning). Emerging research has suggested that these various restrictions, as well as the fear of the virus itself, may have caused children to experience negative mental health consequences (2, 3). In non-pandemic contexts, prevalence studies have shown that between 14 and 25% of children experience psychological distress (4). The profound disruptions in children's normal routines associated with school closures and lockdown measures over several months, in addition to the social isolation and loneliness associated with lockdown measures, pose the risk of additional adverse child mental health outcomes on a population level (5). Some children may be more at risk, such as those with a neurodevelopmental disorder or disability (e.g., attention-deficit/hyperactivity disorder [ADHD], autism spectrum disorder [ASD], cerebral palsy), chronic health condition (e.g., diabetes, obesity), or other pre-existing mental health disorder (e.g., anxiety disorder). Namely, these children may be particularly disadvantaged in facing the effects of the pandemic by virtue of its impacts on access to health care resources and support networks (6–8).

It is thus crucial to clarify the impact of pandemic-associated public health measures on children's mental health and to identify which children are at greater risk of negative outcomes, in order to support them adequately.

## Objectives

The purpose of this study was to examine the impacts of the COVID-19 pandemic on children's mental health. Specifically, the following objectives were pursued: (1) to identify the impacts of the COVID-19 pandemic on the mental health of children between the ages of 5- and 13-years-old, (2) to explore the specific difficulties experienced by children with neurodevelopmental issues or chronic health conditions.

## Methods

The general purpose for conducting a systematic review and meta-analyses is to identify and quantitively summarize the available evidence on a specific aspect of chosen topic; consequently, this methodology was deemed the most appropriate for examining emerging evidence related specifically to the mental health of school-aged children.

### Eligibility Criteria

To be included in this systematic review, studies had to include: (1) quantitative data pertaining to the impacts of the COVID-19 pandemic on the mental health of children aged 5–13 years-old; (2) cross-sectional or longitudinal designs; and (3) original empirical data. Only published peer-reviewed articles were included. Studies with pre and post measures (longitudinal or cross-sectional designs with retrospective measures) were included in the quantitative meta-analyses. Studies using cross-sectional designs with no retrospective measures were summarized for an overview of COVID mental health impacts. Mean age of children in the sample was used to determine study eligibility in each study; mean ages below 5 years old and above 13 years old were excluded.

### Information Sources and Search Strategy

Articles in both French and English, published between January 2020 and June 2021 were searched using the keywords presented in Table 1. Four databases were used: Medline, PsycINFO, ERIC, and Google Scholar. Of note, this study focused solely on the COVID-19 pandemic, as the impacts of other prior pandemic outbreaks (e.g., cholera, flu, HIV, and the plague) were deemed incomparable to the large-scale effects that COVID-19 has brought onto the world.

**Table 1:** Keywords.

**COVID-19**	**Mental health**	**Children**
COVID-19, coronavirus	“Mental health” OR psychosocial OR distress OR wellbeing OR anxiety OR “anxiety disorders” OR mood OR anxious OR stress^*^ OR insomnia OR “mood disorders” OR depressi^*^ OR PTSD OR “post-traumatic stress” OR “suicidal thoughts” OR “suicidal attempts” OR “maladaptive behaviors” OR anger OR confusion OR hopelessness OR panic OR phobia OR “emotional distress” OR inattention OR attention OR agitation OR irritability OR behavior^*^ OR conduct OR hyperactiv^*^ OR “non-compliance” OR psychiatric OR psychological OR “adjustment disorder” OR psychosis OR psychopathology OR “internalized disorder^*^” OR “externalized disorder^*^”	Child^*^ OR pediatric OR pediatric OR youth OR kid^*^ OR juvenile

### Selection of Sources of Evidence

Three research assistants (G.B., T.M., and M.C.) selected the documents; they first reviewed all papers independently, after partial reading (according to title and abstract), then met with the Principal Investigator (C.C.) to discuss disagreements and revise the selection, if necessary. Subsequently, the selection of documents after full reading was carried out by three research assistants (A.M., G.B., and T.M.). When necessary, the Principal Investigator and the research coordinator (E.H.) were consulted to discuss disagreements and revise the selection.

### Data Extraction Process

Three research assistants (A.M., G.B., and T.M.) charted the data from primary studies. The following information was retrieved from primary studies and was noted in the charting grid: study methodology (design, number of participants, and data collection timeline relative to lockdown), country of origin, study population characteristics (age, sex, and children with a disability or a chronic health condition, etc.), study objectives, mental health outcome (e.g., psychological distress, stress, anxiety, depression, and irritability) and on other aspects of health in general (e.g., well-being, physical health, and sleep), as well as quantitative results for calculating effect sizes (*t*-value from paired *t*-test, means and standard deviations, etc.). Data collection dates with respect to COVID-19 lockdown were extracted for each study, which could vary according to country and region. Outcomes were subsequently codified into three mental health categories: internalizing problems, externalizing problems, and sleep disturbances (e.g., insomnia). When studies reported outcomes that were a combination of internalized and externalized problems, or measured wellbeing, they were codified as “mental health.” Sleep duration was treated as a separate category, outside of mental health problems. The extraction grid was developed by the research team and the initial charting of three articles was validated by the co-Principal Investigator (C.M.H). Finally, four authors (CC, EH, CMH, and ELB) read and verified the information from all selected documents.

### Analytic Approach

Random effect meta-analyses were performed using Comprehensive Meta-Analysis (CMA) *3.0* software (9). A positive effect direction was attributed when outcomes indicated a higher score on symptomology (i.e., greater mental health problems), or a lower score on wellbeing and mental health (i.e., worse mental health). For sleep duration, a positive effect size indicated more hours of sleep per night, whereas a negative effect showed fewer hours than before lockdown. For effect size calculation, the pre-post correlation was extracted from studies when available (10). However, most studies did not report it, in which case researchers used a conservative estimate of 0.5 and conducted sensitivity analyses showing little difference between other correlational values (e.g., *r* = 0.1 or 0.9). An overall effect size (Hedges's g) was calculated from the effect sizes of the individual studies, with 95% confidence intervals (*CI*). The random effects approach was used because of the variability in the methodology and measurement scales used between studies (11). A Q-statistic and the *I*^2^ statistics were calculated to quantify heterogeneity between effect sizes (12).

### Subgroup Analyses

Subgroup analyses were conducted based on five variables: (1) type of outcome (externalized, internalized), (2) population (clinical or general), (3) informant for the dependant variable (child or parent), (4) study design (longitudinal or cross-sectional with a retrospective pre-pandemic measure), and (5) the geographical location in which the study was carried out (America, Asia, Europe, and Middle East). Every subgroup analysis was done using study as a unit of analysis to avoid duplication of participants, excepting for the type of outcome, which was done using subgroups within study as a unit of analysis. Continuous variables (e.g., age of the child) could not be tested through meta-regression due to missing data.

### Risk of Bias Across Studies

Publication bias refers to the tendency that studies reporting higher effect sizes are more likely to be published than studies reporting lower effect sizes. Because published literature is more likely to find its way to a meta-analysis, any bias in the literature is likely to be reflected in the meta-analysis (11). To estimate more precisely the possibility of publication bias within our data, a funnel plot was created using the CMA software and the *Trim-and-Fill* Procedure (13).

## Results

### Selection of Sources of Evidence

**Figure 1:**
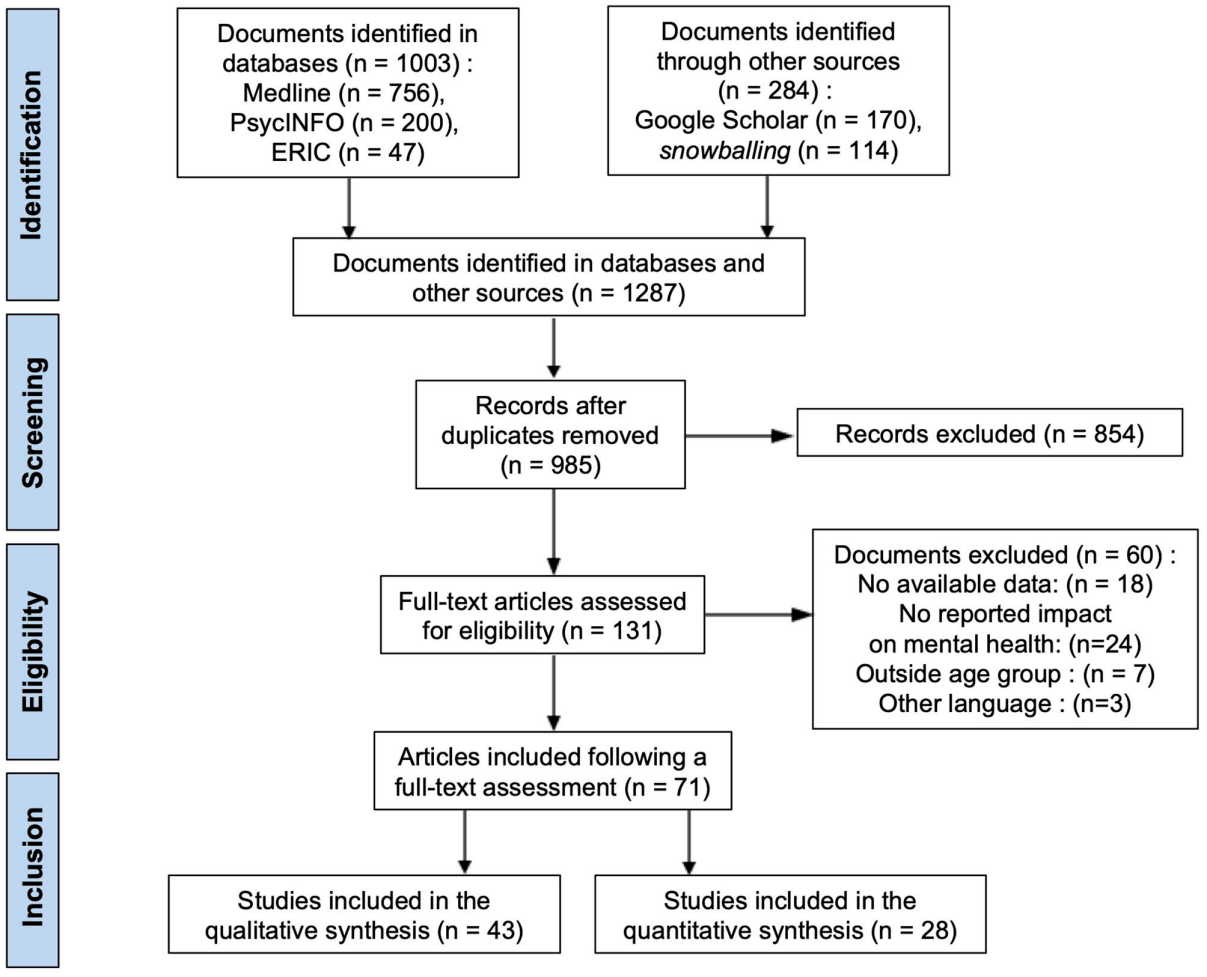
PRISMA flow chart showing the number of included studies for the review.

The search strategy identified 985 documents, and 71 studies were included, 43 in qualitative synthesis (see Supplementary Material) and 28 studies were included in the quantitative synthesis (meta-analysis; see Figure 1).

### Description of Included Studies in the Quantitative Synthesis

Study sample size varied between 12 and 8,124 participants; a total of 14 209 participants were included in the 28 studies. All studies indicated the start of lockdown as being either February [e.g., in China (14)] or March 2020 [e.g., in Italy (15); in Spain (16)] and referred to pre-lockdown as the period before February or March 2020. Studies were conducted in various countries: Italy (*k* = 6), United Kingdom (*k* = 3), Netherlands (*k* = 3), Spain (*k* = 3), Germany (*k* = 1), Switzerland (*k* = 1), these studies were codified as being conducted in “Europe”; China (*k* = 2), Japan (*k* = 2), South Korea (*k* = 1), Singapore (*k* = 1), these studies were codified as being conducted in “Asia”; Israel (*k* = 1), Turkey (*k* = 1) which were coded as “Middle East”; Canada (*k* = 1), United States (*k* = 1) and Argentina (*k* = 1) were coded as “America.”

Meta-analysis study characteristics are presented in Table 2. Among the 28 studies, three studies included a sample of children at-risk for mental health problems or with a neurodevelopmental disorder such as ASD (17, 18), and five included children with medical or physical diagnoses such as epilepsy and obesity (19–22). Two of these studies also had a control group from the general population (17, 23). The other studies were conducted using general population samples (*k* = 20). Among the scales used to measure mental health outcomes, the Strength and Difficulties Questionnaire (SDQ) was used the most frequently (*k* = 7), followed by the Child Behavior Checklist (CBCL; *k* = 3). The cross-sectional studies are presented in Supplementary Material for a brief qualitative synthesis (*k* = 43).

**Table 2:** Characteristics of studies included in the quantitative synthesis.

**Study name**	**Country**	**Population**	***n***	**Age (M; sd)**	**Measure**	**Informant**	**Outcome**	**Design**
Abawi et al. (19)	Netherlands	Obesity	40	10.5	PedsQL	Parent	Int	Longitudinal
Achterberg et al. (24)	Netherlands	General	203	12.0	SDQ	Parent	Ext, Int	Longitudinal
Adegboye et al. (25)	United Kingdom	At risk for MH	142	12.0	SDQ, SCARED	Parent	Ext, Int	Longitudinal
Bentenuto et al. (17)	Italy	NDD	82	7.0	SDQ	Parent	Ext	Retrospective
Bignardi et al. (26)	United Kingdom	General	165	9.9	SDQ, RCADS	Parent	Int, MH health	Longitudinal
Cellini et al. (15)	Italy	General	299	8.0	SDQ	Parent	Ext, Int, Sleep	Retrospective
Chen et al. (14)	China	General	535	10.3	DASS-21	Child	Int	Longitudinal
Choi et al. (27)	South Korea	General	1,236	9–10^*^	KCYWI	Child	Int, Sleep	Longitudinal
Ehrler et al. (23)	Switzerland	Preterm	200	12.8	Kidscreen-27	Parent	MH	Longitudinal
Feinberg et al. (28)	United States	General	108	9.9	SDQ	Parent	Ext, Int	Longitudinal
Francisco et al. (29)	Italy	General	1,480	9.2	–	Parent	Sleep	Retrospective
Ghanamah and Eghbaria-Ghanamah (30)	Israel	General	382	5–11^*^	–	Parent	Sleep	Retrospective
Gimenez-Dasi et al. (31)	Spain	General	75	8.6	SENA	Parent	Ext, Int	Longitudinal
Lim et al. (32)	Singapore	General	336	8.0	–	Parent	Sleep	Retrospective
Logan et al. (20)	Canada	NeuroInf	87	13.0	–	Child	Sleep	Longitudinal
López-Bueno et al. (33)	Spain	General	459	9.6	–	Parent	Sleep	Retrospective
Medrano et al. (16)	Spain	General	113	12.0	–	Child	Sleep	Longitudinal
Morgül et al. (34)	United Kingdom	General	927	7.5	–	Parent	Sleep	Retrospective
Pasca et al. (21)	Italy	Epilepsy	23	13.0	CBCL	Parent	Int	Longitudinal
Saito et al. (35)	Italy	General	135	11.4	WHO-5-J	Child	MH	Longitudinal
Schnaiderman et al. (36)	Argentina	General	267	11.1	–	Parent	Sleep	Retrospective
Siracusano et al. (18)	Italy	ASD, Sotos	12	8.5	CBCL	Parent	Ext	Longitudinal
Takahashi and Honda (37)	Japan	General	4,738	12.4	SDQ	Parent	Ext, Int	Longitudinal
Tanir et al. (38)	Turkey	General	61	13.6	CY-BOCS	Parent	MH	Longitudinal
van Gorp et al. (22)	Netherlands	Oncology	407	4–18^*^	PedsQL	Parent, child	Int	Longitudinal
Wunsch et al. (39)	Germany	General	752	4–17^*^	Kidscreen-27	Parent	MH	Longitudinal
Xiang et al. (40)	China	General	875	6–12^*^	CDI-S	Parent	Int	Longitudinal
Zaccaria et al. (41)	Italy	General	70	9.1	CBCL	Parent	Ext, Int	Longitudinal

* *Age range was provided for studies that did not report mean and standard deviation; ASD, Autism Spectrum Disorder; NDD, Neurodevelopmental Disorders; NeuroInf, Neuroinflammatory; CBCL, Child Behavior Checklist; CDI-S, Child Depression Inventory—Short Form; DASS-21, Depression Anxiety Stress Scale-21; KCYWI, Korean Children and Youth Wellbeing Index; MH, Mental Health; PedsQL, Pediatric Quality of Life Inventory; RCADS, Revised Children's Anxiety and Depression Scale; SCARED, Screen for Child Anxiety Related Disorders; SDQ, Strength and Difficulties Questionnaire; SENA, System of Evaluation of Children and Adolescent*.

### Main Analysis

Among the 28 included longitudinal or retrospective studies, a series of two meta-analyses was conducted with studies which included mental health outcomes (internalizing, externalizing, and sleep disturbances) (*k* = 21) and studies including sleep duration (*k* = 9).

### Studies Including Mental Health Outcomes

Across all studies with mental health outcomes (*k* = 21), a small overall effect size of *g* = 0.276 (95% CI [0.15, 0.41]; *p* < 0.001) was observed for children's mental health before and during lockdown. This effect indicates an overall worsening of mental health in children across different outcomes (internalizing and externalizing symptoms, well-being, and sleep disturbances), but of small magnitude. As the *Q* statistics is significant (*Q* = 1,008,807, *p* < 0.001), it is pertinent to conduct moderator analysis. Table 3 presents subgroup analyses for mental health studies.

**Table 3:** Subgroup analyses for mental health studies (*k* = 21).

**Variables**	***k***	***N***	***g***	**Confidence interval**		**Contrast Q' (*p*)**
				**Lower limit**	**Upper limit**	
**All studies**	21	10,425	0.28^***^	0.15	0.41	
**Mental health problems**						
Internalizing	19	8,916	0.21^**^	0.06	0.37	
Externalizing	15	5,729	0.14^***^	0.08	0.21	
**Population**						
Clinical	4	836	0.15	−0.10	0.40	
General	16	10,039	0.30^***^	0.14	0.45	
Clinical vs. General						3.60 (0.17)
**Design**						
Longitudinal	18	9,777	0.27^**^	0.11	0.43	
Retrospective	3	648	0.27^*^	0.15	0.39	
Longitudinal vs. Retrospective						0.00 (0.97)
**Informant**						
Child	3	1,906	0.57			
Parent	17	8,112	0.23^***^			
Both	1	407	0.07			
Child vs. parent						*5.16* (*0.076*)^†^
**Geographical location**						
Asia	3	6,849	0.06			
Europe	15	3,140	0.31^**^			
America	2	375	0.14			
Middle East	1	61	0.76^***^			
Asia vs. Europe						4.7 (0.03)

* *p < 0.05*;

** *p < 0.01*;

*** *p < 0.001*;

† *p < 0.08*.

*k, Number of studies; N, Number of participants; g, Hedge's g effect size*.

#### Type of Outcome

A small effect size was observed for internalizing symptoms (*g* = 0.215; 95% CI [0.06, 0.37]; *p* < 0.001; *k* = 19) and for externalizing symptoms (*g* = 0.141 (95% CI [0.08, 0.21]; *p* < 0.01; *k* = 15), showing that children demonstrated higher levels of both internalized and externalized symptoms during lockdown. Considering that some participants may be duplicated in these analyses, a contrast analysis was not done to determine whether these two effect sizes are statistically different one from another, as recommended by Lipsey and Wilson (10).

#### Population Type

Children with a clinical condition (e.g., ADHD, ASD, and epilepsy) did not seem to be affected differently in comparison with children from the general population (*Contrast Q*' = 3.60; *p* = 0.166).

#### Study Design

Effect sizes reported by studies using a longitudinal design (*g* = 0.27; 95% CI [0.11, 0.44]; *p* < 0.01; *k* = 18) did not differ from cross-sectional designs using retrospective measures for the pre pandemic measure (*g* = 0.27; 95% CI [0.15, 0.39]; *p* < 0.001; *k* = 3; *Contrast Q*'= 0.002; *p* = 0.97).

#### Informant

There were larger effect sizes in studies where the child was the informant (*g* = 0.57; 95% CI [−0.36, 1.50]; *p* = 0.23; *k* = 3) in comparison to studies in which the parent as the informant (*g* = 0.23; 95% CI [−0.11, 0.35]; *p* < 0.001; *k* = 17). However, this result is based on a limited number of studies (only three studies had children reporting data) and should be interpreted with caution.

#### Country

Changes in children's mental health were three times larger for European countries (*g* = 0.31; 95% CI [0.10, 0.52]; *p* < 0.01; *k* = 15) in comparison to Asian countries (*g* = 0.01; 95% CI [−0.04, 0.15]; *p* = 0.26; *k* = 3). This difference is statistically significant (*Contrast Q*' = 4.7; *p* = 0.03).

### Studies Including Sleep Duration Outcomes

Children's sleep was significantly affected during the COVID-19 lockdown period, with significantly longer sleeping hours during lockdown compared to before (*g* = 0.324; 95% CI [0.10, 0.55]; *p* = 0.004; *k* = 9). Subgroup analysis was not performed due to small number of studies in each subset (<4), according to best practices in meta-analysis (42).

### Heterogeneity and Publication Bias

The *Q*-test for heterogeneity was significant (*Q* = 1,008,807, *p* < 0.001). The *I*^2^ statistic was used in complement to the Q statistic to quantify the degree of heterogeneity in the aggregated studies, as proposed by Huedo-Medina et al. (43). The *I*^2^, which represents the percentage of variation across studies that is due to heterogeneity, was 98.02.

**Figure 2:**
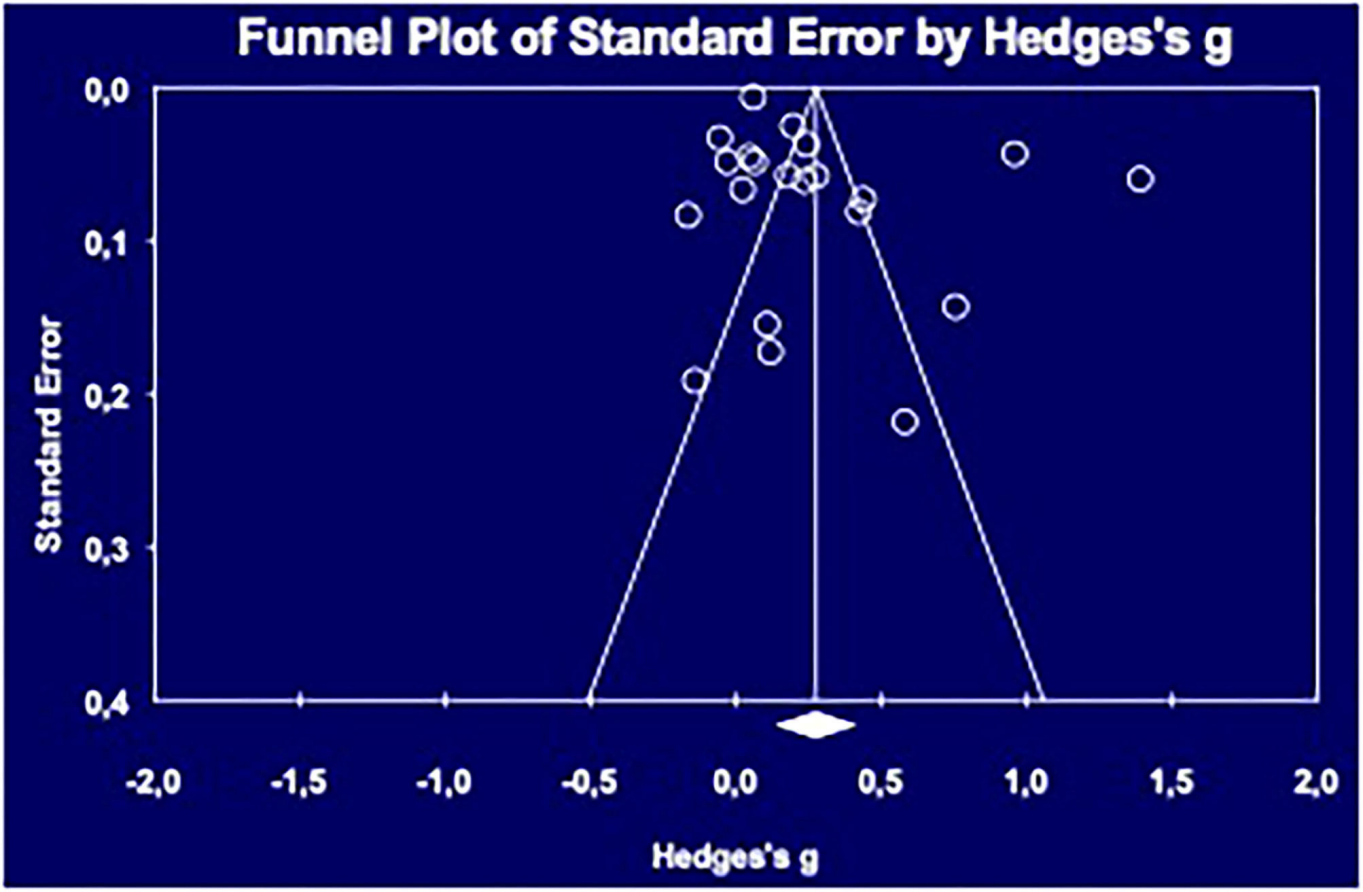
Funnel Plot for publication bias.

Visual observation of the funnel plot (Figure 2) does not show the presence of a publication bias, which is confirmed by the *Trim-and-Fill* procedure. No outlier was observed in this meta-analysis.

## Discussion

This meta-analysis aimed to identify the impacts of the COVID-19 pandemic on children's mental health and to explore the specific issues experienced by children with neurodevelopmental issues or chronic health conditions. Overall, the quantitative synthesis of longitudinal and retrospective studies suggests that a negative impact of the pandemic was observed on children's mental health, but this impact is of weak magnitude, for both internalized (e.g., anxiety or depression) and externalized symptoms (e.g., conduct disorder, hyperactivity). These results support, to an extent, the large body of cross-sectional studies that have collected data during the pandemic (without pre-pandemic measures) and which report consistently negative outcomes regarding children's mental health during the pandemic. The cross-sectional studies identified in the qualitative section of this review suggest similar conclusions, with a majority of studies (35/43) reporting negative an overall negative impact on mental health associated with the COVID-19 lockdown.

Similarly, recently published systematic reviews examining the impact of the pandemic on children's mental health suggest that the lockdown associated with Covid-19 pandemic had a negative impact on children' mental health (44–46). However, the cross-sectional nature of the included studies makes it difficult to draw firm conclusions from these reviews. The present meta-analysis is the first to provide an estimate of the changes in children's mental health symptoms during Covid-19 pandemic by way of a meta-analysis including longitudinal data. Our meta-analysis suggests that the COVID-19 period was associated with weak impact on children's mental health, which contrasts with a recent meta-analysis that has looked at the prevalence of anxiety and depression among children and teenagers during the pandemic (47). These authors have synthesized prevalence data reported in cross-sectional studies and have observed a prevalence of anxiety (20.5%) and depression (25.2%) two times higher than prepandemic estimates.

If we look specifically at the well-being of children having special needs, the conclusions remain the same. Indeed, a subgroup analysis comparing clinical samples and general population samples revealed that having a neurodevelopmental disorder or chronic health condition did not place these children at higher risk of developing mental health symptoms with to the COVID-19 pandemic lockdown measures. This conclusion may seem surprising, considering that they stand in contrast to Panda et al.'s (46) systematic review's conclusions. Given that our results arise from a small subset of studies with mental health outcomes (four studies included clinical samples), and thus need to be interpreted with caution. In addition, these four samples are very heterogeneous and include children with epilepsy, ASD, obesity, who were both preterm and with other neurodevelopmental disorders. As some authors have highlighted, children with psychiatric or neurodevelopmental disorders do not necessary show homogeneous responses to the pandemic (48). For example, Cost et al. (48) observed that whereas those with social anxiety or learning disorders showed reduced mental health symptoms (reduced anxiety and irritability), those with ADHD or ASD showed greater irritability and lower mood. These authors hypothesized that children with anxiety or learning disorders might have felt relief from the lockdown situation, while those with ADHD or ASD most likely suffered from a loss of structure and fewer social interactions (48). In a future meta-analysis including a larger number of studies, clinical samples could be grouped to achieve more homogeneous subgroups.

Moreover, this meta-analysis shows a trend toward a larger effect size when mental health effects are self-reported by children themselves. This result should be interpreted with caution given the small number of studies with self-report measures but could be the object of further studies with larger samples sizes to account for children's perspectives on changes in their own mental health.

Finally, the impact of the pandemic on children's mental health is three times larger in studies conducted in European countries, in comparison with studies conducted in Asian Countries. This result does not corroborate the conclusions reported in other reviews, and in fact is at the opposite of conclusions stated by Panda et al. (46) who observed a higher prevalence of psychological morbidities in Asian countries in comparison to European countries like Spain. Once again, caution is warranted in drawing conclusions from our subgroup analysis since this analysis is based on only three studies conducted in Asian countries.

### Sleep Duration

Our study revealed significant changes in sleep duration during the COVID pandemic. The clinical significance of these changes remains unknown given that sleep can be both an indicator of healthy lifestyle behaviors and poor mental health (e.g., in the case of a depressive disorder). Additional studies are needed focusing on sleep quality instead of sleep duration, to draw conclusions on the associations between the COVID-19 pandemic and children's sleep-related outcomes.

### Limitations

Although this meta-analytic review includes longitudinal data that allows to shed light on the impact of the pandemic on children's mental health, most studies relied on parents' perception about their children's psychological state. As parental distress may interfere with their report of their children's functioning, future work in which the perspective of children is incorporated is strongly recommended, especially with preliminary data from this meta-analysis suggesting that effect sizes reported by children could be larger than parent's report. Another limitation of the present meta-analysis is the heterogeneity in the measurement tools used in the primary studies included. In addition, it must be highlighted that these instruments allow to quantify symptoms but not to concern clinical diagnosis. Finally, another limitation is the fact that the data collections in included studies varied from 3 weeks to 6 months with respect to the implementation of public health measures; therefore, it is difficult to confirm a direct link between the application of the measures and children's mental health. Rigorous, population-based longitudinal studies correlated with public health measures, using large samples and standardized tools would address these gaps. Finally, future research should include longer follow-ups to address the long-term consequences of the Covid-19 pandemic on children's mental health.

## Conclusion

In sum, studies included in this meta-analysis suggest that changes seen in children's mental health during the early phases of the COVID-19 pandemic period, which included the application of several public health measures were relatively small. More research is needed to improve our understanding of the long-term impacts of the COVID-19 pandemic on children's mental health, especially with regards to the identification of protective factors found in children who may have been less affected by the pandemic. Several questions remain unanswered including which characteristics of living environments can positively or negatively affect children's capacity to adapt to major public health crises such as the COVID-19 pandemic. Future research should include self-reported measures completed by children themselves, and follow not only mental health outcomes through time, but also developmental, learning, academic and eventually work-related outcomes as the pandemic and post-pandemic period unfolds. It is crucial to develop a better understanding of children's psychological needs during this pandemic, to elaborate comprehensive and evidence-based interventions to support children and their families through these unprecedented times.

## Data Availability Statement

The raw data supporting the conclusions of this article will be made available by the authors, without undue reservation.

## Author Contributions

E-LB wrote the manuscript and took part in all steps of the research project. CM-H made substantial revisions to the manuscript. CC and CM-H were co-PIs on this project and obtained funding for it. AM contributed to the analyses and in writing the manuscript. TM, EH, NC, MM, MG, and the PRISME-COVID Team revised the submitted manuscript and were involved in all steps of the research project. All authors contributed to the article and approved the submitted version.

## Funding

This study was supported by CIHR (No. 171716).

## Conflict of Interest

The authors declare that the research was conducted in the absence of any commercial or financial relationships that could be construed as a potential conflict of interest.

## Publisher's Note

All claims expressed in this article are solely those of the authors and do not necessarily represent those of their affiliated organizations, or those of the publisher, the editors and the reviewers. Any product that may be evaluated in this article, or claim that may be made by its manufacturer, is not guaranteed or endorsed by the publisher.

## PRISME-COVID Team

Chantal Camden, Catherine Malboeuf-Hurtubise, Pasquale Roberge, Mélissa Généreux, Julie Lane, Emmanuelle Jasmin, Jean-Claude Kalubi, Eve-Line Bussiéres, Nicholas Chadi, Marjorie Montreuil, Karen Hurtubise, Grégory Bach, Trinity Mastine, Marie Chrysagis, Marie-Pier Turner, Cheyenne Gauvin, Élodie Hérault.
