# Supplementary material for: Consequences of the COVID-19 Pandemic on Children's Mental Health: A Meta-Analysis
Source: Front Psychiatry. 2021 Dec 1;12:691659. doi: 10.3389/fpsyt.2021.691659 (PMC8672800; doi:10.3389/fpsyt.2021.691659)
Supplement: Supplementary file 1 [file Table_1.pdf]

Supplementary material

Transversal studies reporting on the impact of COVID-19 on mental health

Study Name	Population	n	Age	Outcome	Impact on mental health	Results
Alves et al., 2021	Obesity	64	11.8 (1.2)	Internalizing	No impact	Anxiety and emotions did not change
Bacaro et al., 2021	General	2361	8.1 (2.62)	Internalizing Sleep	Negative	High rate of insomnia symptoms associated with negative mood
Baudry et al., 2021	General	237	10.15 (3.2)	Internalizing	Negative	High rates of anxiety and depression
Berard et al., 2021	ASD	239	9.11 (4)	Externalizing Sleep	Negative	More challenging behaviors and sleep difficulties
Castro-Kemp et al., 2021	Special needs	83	10.5 (3.87)	Mental health	Negative	Most parents reported a detrimental impact of school closure
Colizzi et al., 2020	ASD	527	13 (8.1)	Externalizing	Negative	Previous behavior problems predicted greater disruptive behavior during lockdown
Cusinato et al., 2020	General	463	8.22 (3.96)	Mental health	Negative	Low mental health and high rates of hyperactivity
Davico et al., 2021	General	2419	12.3 (3.2)	Internalizing	Negative	High rates of psychological distress
Dilek et al., 2021	Multiple sclerosis	30	8-10*	Internalizing	Negative	High depression and anxiety symptoms in clinical and control group
Drouin et al., 2020	General	260	7.69 (5.64)	Internalizing	Negative	High proportion of sample showing moderate to severe anxiety
Duan et al., 2020	General	359	7-12*	Internalizing	Negative	Higher levels of depression and anxiety
Fitzpatrick et al., 2020	General	56	8.21 (4.94)	Internalizing Externalizing	Negative	High levels of anxiety, depression, and problem behaviors
Gadermann et al., 2021	General	618	0-18*	Mental health	No impact	No change in mental health but more conflict and feelings of closeness
Garcia de Avila et al., 2020	General	289	8.84 (2.05)	Internalizing	Negative	Higher prevalence of anxiety compared to norms
Garcia-Adasme et al., 2021	General	2292	0-17*	Internalizing	Negative	Higher anxiety and more somatic complaints
Guller et al., 2021	NDD	299	10.32 (4.57)	Internalizing Externalizing Sleep	Negative	More emotional and behavioral problems, no change in sleep problems compared to past literature

Supplementary material

Transversal studies reporting on the impact of COVID-19 on mental health

Hassan et al., 2021	Hematology Oncology	292	8.72 (3.66)	Internalizing	Negative	High levels of anxiety, which were associated with lower quality of life
Lavigne-Cervan et al., 2021	General	1028	10.34 (3.64)	Internalizing Sleep	Negative	Higher levels of anxiety and poor sleep quality
Liang et al., 2020	General	1074	8.99 (1.97)	Internalizing	Negative	High levels of anxiety and negative mood
Liu et al., 2021a	General	1264	9.81	Internalizing Externalizing	No impact	Same prevalence than before lockdown
Ma et al., 2021	General	668	7-15*	Internalizing	Negative	High prevalence of post-traumatic and depressive symptoms
Mumbardo-Adam et al., 2021	ASD	47	7.3 (3.4)	Internalizing	Positive	Good adaptation, happier than before although more irritable
Nonweiler et al., 2020	NDD	453	4-15*	Internalized Externalized	Negative	Worse emotional symptoms and hyperactivity than before
Nunez et al., 2021	ASD	152	6**	Externalized	Negative	Behavioral difficulties increased in intensity or frequency
Orgiles et al., 2021a	General	515	3-18*	Internalizing	Negative	Higher anxiety and depression scores
Qin et al., 2021	General	1 199 320	12.04 (3.01)	Internalizing	Negative	Higher prevalence of psychological distress
Romero et al., 2020	General	940	7.29 (2.39)	Internalized Externalized	Negative	More emotional problems, conduct problems and hyperactivity
Saddik et al., 2021	General	2200	0-18*	Internalizing	Negative	More anxiety and emotional problems
Sama et al., 2021	General	310	0-18*	Mental health	Negative, No impact	More irritation and anger, sleep problems. No changes in anxiety and depression
Schmidt et al., 2021	General	5823	7.3 (4.3)	Internalizing	Negative	More anxiety, depression, aggressive problems
Segre et al., 2021	General	82	10.4	Internalizing Sleep	Negative	More psychological distress and disturbed sleep routine
Spinelli et al., 2020a	General	810	7.16 (3.34)	Internalizing	No impact	No change in emotion regulation abilities
Swansburg et al., 2021	ADHD	587	10.14 (3.06)	Internalizing Externalizing	Negative	High prevalence of anxiety and depression
Tang et al., 2021	General	4391	11.86 (2.32)	Internalizing	Negative	Higher anxiety levels

Supplementary material

Transversal studies reporting on the impact of COVID-19 on mental health

Tso et al., 2020	General	17029	8.51 (1.86)	Mental health	No impact	Little impact on psychological difficulties
Uccella et al., 2021	General	3245	0-18*	Internalizing Externalizing Sleep habits	Negative	More difficulties falling asleep, waking up, short breath, more inatttention
Ueda et al., 2021	NDD	136	10.6 (2.6)	Internalizing Externalizing	Negative	High levels of internalizing and externalizing problems
Verlenden et al., 2021	General	1290	5-12*	Mental health	No impact	Parents reported no change or better mental health, especially if hybrid or in-person schooling
Xie et al., 2020	General	1784	5-12*	Internalizing	Negative	High anxiety and depression
Yue et al., 2020	General	1360	10.56 (1.79)	Internalizing	No impact	Did not suffer from major psychological distress
Zainel et al., 2021	General	6608	7-18*	Internalizing Sleep	Negative	More sleep problems and anger
Zhao et al., 2020	General	2010	11 (1.7)	Internalizing Externalizing Sleep	Negative	Greater emotional and behavioral problems
Zhu et al., 2021	General	2863	12.6 (1.3)	Mental health	Negative	negative impact on mental health but positive changes in routine

*Studies reporting only age range without mean and standard deviation; **median

Supplementary material

Transversal studies reporting on the impact of COVID-19 on mental health

Alves, J. M., Yunker, A. G., DeFendis, A., Xiang, A. H., & Page, K. A. (2021). BMI status and associations between affect, physical activity and anxiety among U.S. children during COVID-19. *Pediatric obesity*, e12786-e12786. https://doi.org/10.1111/ijpo.12786

Bacaro, V., Chiabudini, M., Buonanno, C., De Bartolo, P., Riemann, D., Mancini, F., & Baglioni, C. (2021). Sleep characteristics in Italian children during home confinement due to COVID-19 outbreak. *Clinical Neuropsychiatry: Journal of Treatment Evaluation, 18*(1), 13-27. https://doi.org/10.36131/ cnfioritieditore20210102

Baudry, C., Pearson, J., Massé, L., Ouellet, G., Bégin, J.-Y., Couture, C., Gilbert, E., Slater, E., & Burton, K. (2021). Psychosocial adaptation and mental health of young people living in the context of a COVID-19 pandemic in Quebec, Canada. Descriptive and preliminary data. *Canadian Psychology/Psychologie canadienne*. https://doi.org/10.1037/cap0000271

Berard, M., Rattaz, C., Peries, M., Loubersac, J., Munir, K., & Baghdadli, A. (2021). Impact of containment and mitigation measures on children and youth with ASD during the COVID-19 pandemic: Report from the ELENA cohort. *Journal of psychiatric research, 137*, 73-80. https://doi.org/10.1016/j.jpsychires.2021.02.041

Castro-Kemp, S., & Mahmud, A. (2021). School closures and returning to school: views of parents of children with disabilities in England during the Covid-19 pandemic. *Frontiers in Education, 6*, Article 666574. https://doi.org/10.3389/feduc

Colizzi, M., Sironi, E., Antonini, F., Ciceri, M. L., Bovo, C., & Zoccante, L. (2020). Psychosocial and behavioral impact of COVID-19 in autism spectrum disorder: An online parent survey. *Brain Sciences, 10(6),* 341. https://doi.org/10.3390/brainsci10060341

Cusinato, M., Iannattone, S., Spoto, A., Poli, M., Moretti, C., Gatta, M., & Miscioscia, M. (2020). Stress, Resilience, and Well-Being in Italian Children and Their Parents during the COVID-19 Pandemic. *International journal of environmental research and public health, 17*(22). https://doi.org/10.3390/ijerph17228297

Davico, C., Ghiggia, A., Marcotulli, D., Ricci, F., Amianto, F., & Vitiello, B. (2021). Psychological Impact of the COVID-19 Pandemic on Adults and Their Children in Italy. *Frontiers in Psychiatry, 12*, 572997-572997. https://doi.org/10.3389/fpsyt.2021.572997

Dilek, T. D., Boybay, Z., Kologlu, N., Tin, O., Güler, S., & Saltık, S. (2021). The impact of SARS-CoV2 on the anxiety levels of subjects and on the anxiety and depression levels of their parents. *Multiple sclerosis and related disorders, 47*, 102595-102595. https://doi.org/10.1016/j.msard.2020.102595

Drouin, M., McDaniel, B. T., Pater, J., & Toscos, T. (2020). How Parents and Their Children Used Social Media and Technology at the Beginning of the COVID-19 Pandemic and Associations with Anxiety. *Cyberpsychology, behavior and social networking, 23*(11), 727-736. https://doi.org/10.1089/cyber.2020.0284

Supplementary material

Transversal studies reporting on the impact of COVID-19 on mental health

Duan, L., Shao, X., Wang, Y., Huang, Y., Miao, J., Yang, X., & Zhu, G. (2020). An investigation of mental health status of children and adolescents in china during the outbreak of COVID-19. *Journal of affective disorders, 275*, 112-118. https://doi.org/10.1016/j.jad.2020.06.029

Fitzpatrick, O., Carson, A., & Weisz, J. R. (2020). Using Mixed Methods to Identify the Primary Mental Health Problems and Needs of Children, Adolescents, and Their Caregivers during the Coronavirus (COVID-19) Pandemic. *Child Psychiatry and Human Development*. https://doi.org/10.1007/s10578-020-01089-z

Gadermann, A. C., Thomson, K. C., Richardson, C. G., Gagné, M., McAuliffe, C., Hirani, S., & Jenkins, E. (2021). Examining the impacts of the COVID-19 pandemic on family mental health in Canada: findings from a national cross-sectional study. *BMJ open, 11*(1), e042871-e042871. https://doi.org/10.1136/bmjopen-2020-042871

Garcia de Avila, M. A., Hamamoto Filho, P. T., Jacob, F. L. d. S., Alcantara, L. R. S., Berghammer, M., Jenholt Nolbris, M., Olaya-Contreras, P., & Nilsson, S. (2020). Children's Anxiety and Factors Related to the COVID-19 Pandemic: An Exploratory Study Using the Children's Anxiety Questionnaire and the Numerical Rating Scale. *International journal of environmental research and public health, 17*(16). https://doi.org/10.3390/ijerph17165757

Garcia-Adasme, S. I., Cárdenas-Rebollo, J. M., Jimenez-Perianes, A., Lalinde, M., Jimeno, S., Ventura, P. S., Díaz, A., & López-Escobar, A. (2021). Pediatric home confinement due to COVID-19: Somatic and anxiety spectrum consequences. *Journal of Clinical Nursing*. https://doi.org/10.1111/jocn.15829

Guller, B., Yaylaci, F., & Eyuboglu, D. (2021). Those in the shadow of the pandemic: impacts of the COVID-19 outbreak on the mental health of children with neurodevelopmental disorders and their parents. *International Journal of Developmental Disabilities*. https://doi.org/10.1080/20473869.2021.1930827

Hassan, E., Nagui Rizk, D., Aly, N. M., & El Chazli, Y. (2021). Impact of the COVID-19 pandemic on health-related quality of life and anxiety in pediatric hematology/oncology patients. *Pediatric hematology and oncology*, 1-15. https://doi.org/10.1080/08880018.2021.1922559

Lavigne-Cerván, R., Costa-López, B., Juárez-Ruiz de Mier, R., Real-Fernández, M., Sánchez-Muñoz de León, M., & Navarro-Soria, I. (2021). Consequences of COVID-19 Confinement on Anxiety, Sleep and Executive Functions of Children and Adolescents in Spain. *Frontiers in psychology, 12*, 565516-565516. https://doi.org/10.3389/fpsyg.2021.565516

Liang, Z., Delvecchio, E., Buratta, L., & Mazzeschi, C. (2020). “Ripple effect”: Psychological responses and coping strategies of Italian children in different COVID-19 severity areas. *Revista de Psicología Clínica con Niños y Adolescentes, 7*(3), 49-58. https://doi.org/10.21134/rpcna.2020.mon.2054

Liu, Q., Zhou, Y., Xie, X., Xue, Q., Zhu, K., Wan, Z., Wu, H., Zhang, J., & Song, R. (2021). The prevalence of behavioral problems among school-aged children in home quarantine during the

COVID-19 pandemic in china. *Journal of affective disorders, 279*, 412-416. https://doi.org/10.1016/j.jad.2020.10.008

Ma, Z., Idris, S., Zhang, Y., Zewen, L., Wali, A., Ji, Y., Pan, Q., & Baloch, Z. (2021). The impact of COVID-19 pandemic outbreak on education and mental health of Chinese children aged 7–15 years: an online survey. *BMC pediatrics, 21*(1). https://doi.org/10.1186/s12887-021-02550-1

Mumbardó-Adam, C., Barnet-López, S., & Balboni, G. (2021). How have youth with Autism Spectrum Disorder managed quarantine derived from COVID-19 pandemic? An approach to families perspectives. *Research in Developmental Disabilities, 110*, 103860-103860. https://doi.org/10.1016/j.ridd.2021.103860

Nonweiler, J., Rattray, F., Baulcomb, J., Happé, F., & Absoud, M. (2020). Prevalence and Associated Factors of Emotional and Behavioural Difficulties during COVID-19 Pandemic in Children with Neurodevelopmental Disorders. *Children (Basel, Switzerland), 7*(9). https://doi.org/10.3390/children7090128

Nuñez, A., Le Roy, C., Coelho-Medeiros, M. E., & López-Espejo, M. (2021). Factors affecting the behavior of children with ASD during the first outbreak of the COVID-19 pandemic. *Neurological sciences : official journal of the Italian Neurological Society and of the Italian Society of Clinical Neurophysiology, 42*(5), 1675-1678. https://doi.org/10.1007/s10072-021-05147-9

Orgilés, M., Espada, J. P., Delvecchio, E., Francisco, R., Mazzeschi, C., Pedro, M., & Morales, A. (2021). Anxiety and Depressive Symptoms in Children and Adolescents during COVID-19 Pandemic: A Transcultural Approach. *Psicothema, 33*(1), 125-130. https://doi.org/10.7334/psicothema2020.287

Qin, Z., Shi, L., Xue, Y., Lin, H., Zhang, J., Liang, P., Lu, Z., Wu, M., Chen, Y., Zheng, X., Qian, Y., Ouyang, P., Zhang, R., Yi, X., & Zhang, C. (2021). Prevalence and Risk Factors Associated With Self-reported Psychological Distress Among Children and Adolescents During the COVID-19 Pandemic in China. *JAMA Network Open, 4*(1), e2035487-e2035487. https://doi.org/10.1001/jamanetworkopen.2020.35487

Romero, E., López-Romero, L., Domínguez-Álvarez, B., Villar, P., & Gómez-Fraguela, J. A. (2020). Testing the Effects of COVID-19 Confinement in Spanish Children: The Role of Parents' Distress, Emotional Problems and Specific Parenting. *International journal of environmental research and public health, 17*(19). https://doi.org/10.3390/ijerph17196975

Saddik, B., Hussein, A., Albanna, A., Elbarazi, I., Al-Shujairi, A., Temsah, M.-H., Sharif-Askari, F. S., Stip, E., Hamid, Q., Halwani, R., Saheb Sharif-Askari, F., Stip, E., Hamid, Q., & Halwani, R. (2021). The psychological impact of the COVID-19 pandemic on adults and children in the United Arab Emirates: a nationwide cross-sectional study. *BMC psychiatry, 21*(1). https://doi.org/10.1186/s12888-021-03213-2

Supplementary material

Transversal studies reporting on the impact of COVID-19 on mental health

Sama, B. K., Kaur, P., Thind, P. S., Verma, M. K., Kaur, M., & Singh, D. D. (2021). Implications of COVID-19-induced nationwide lockdown on children's behaviour in Punjab, India. *Child: care, health and development*, *47*(1), 128-135. https://doi.org/10.1111/cch.12816

Schmidt, S. J., Barblan, L. P., Lory, I., & Landolt, M. A. (2021). Age-related effects of the COVID-19 pandemic on mental health of children and adolescents. *European journal of psychotraumatology, 12*(1), 1901407-1901407. https://doi.org/10.1080/20008198.2021.1901407

Segre, G., Campi, R., Scarpellini, F., Clavenna, A., Zanetti, M., Cartabia, M., & Bonati, M. (2021). Interviewing children: the impact of the COVID-19 quarantine on children's perceived psychological distress and changes in routine. *BMC pediatrics*, *21*(1). https://doi.org/10.1186/s12887-021-02704-1

Spinelli, M., Lionetti, F., Setti, A., & Fasolo, M. (2020). Parenting Stress During the COVID-19 Outbreak: Socioeconomic and Environmental Risk Factors and Implications for Children Emotion Regulation. *Family Process*. https://doi.org/10.1111/famp.12601

Swansburg, R., Hai, T., MacMaster, F. P., & Lemay, J.-F. (2021). Impact of COVID-19 on lifestyle habits and mental health symptoms in children with attention-deficit/hyperactivity disorder in Canada. *Paediatrics & Child Health*. https://doi.org/10.1093/pch/pxab030

Tang, S., Xiang, M., Cheung, T., & Xiang, Y.-T. (2021). Mental health and its correlates among children and adolescents during COVID-19 school closure: The importance of parent-child discussion. *Journal of affective disorders, 279,* 353-360. https://doi.org/10.1016/j.jad.2020.10.016

Tso, W. W. Y., Wong, R. S., Tung, K. T. S., Rao, N., Fu, K. W., Yam, J. C. S., Chua, G. T., Chen, E. Y. H., Lee, T. M. C., Chan, S. K. W., Wong, W. H. S., Xiong, X., Chui, C. S., Li, X., Wong, K., Leung, C., Tsang, S. K. M., Chan, G. C. F., Tam, P. K. H., Chan, K. L., Kwan, M. Y. W., Ho, M. H. K., Chow, C. B., Wong, I. C. K., & Lp, P. (2020). Vulnerability and resilience in children during the COVID-19 pandemic. *European Child & Adolescent Psychiatry*. https://doi.org/10.1007/s00787-020-01680-8

Uccella, S., De Grandis, E., De Carli, F., D'Apruzzo, M., Siri, L., Preiti, D., Di Profio, S., Rebora, S., Cimellaro, P., Biolcati Rinaldi, A., Venturino, C., Petralia, P., Ramenghi, L. A., & Nobili, L. (2021). Impact of the COVID-19 Outbreak on the Behavior of Families in Italy: A Focus on Children and Adolescents. *Frontiers in Public Health, 9,* 608358-608358. https://doi.org/10.3389/fpubh.2021.608358

Ueda, R., Okada, T., Kita, Y., Ozawa, Y., Inoue, H., Shioda, M., Kono, Y., Kono, C., Nakamura, Y., Amemiya, K., Ito, A., Sugiura, N., Matsuoka, Y., Kaiga, C., Kubota, M., & Ozawa, H. (2021). The quality of life of children with neurodevelopmental disorders and their parents during the Coronavirus disease 19 emergency in Japan. *Scientific reports, 11*(1), 3042-3042. https://doi.org/10.1038/s41598-021-82743-x

Supplementary material

Transversal studies reporting on the impact of COVID-19 on mental health

Verlenden, J. V., Pampati, S., Rasberry, C. N., Liddon, N., Hertz, M., Kilmer, G., Viox, M. H., Lee, S., Cramer, N. K., Barrios, L. C., & Ethier, K. A. (2021). Association of Children's Mode of School Instruction with Child and Parent Experiences and Well-Being During the COVID-19 Pandemic - COVID Experiences Survey, United States, October 8-November 13, 2020. *MMWR. Morbidity and mortality weekly report*, *70*(11), 369-376. https://doi.org/10.15585/mmwr.mm7011a1

Xie, X., Xue, Q., Zhou, Y., Zhu, K., Liu, Q., Zhang, J. & Song, R. (2020). Mental health status among children in home lockdown during the coronavirus disease 2019 outbreak in Hubei Province, China. *JAMA Pediatrics, 174(9),* 898–900. https://doi.org/10.1001/jamapediatrics.2020.1619

Yue, J., Zang, X., Le, Y., & An, Y. (2020). Anxiety, depression and ptsd among children and their parent during 2019 novel coronavirus disease (covid-19) outbreak in china. *Current Psychology: A Journal for Diverse Perspectives on Diverse Psychological Issues*, No Pagination Specified-No Pagination Specified. https://doi.org/10.1007/s12144-020-01191-4

Zainel, A. A., Qotba, H., Al-Maadeed, A., Al-Kohji, S., Al Mujalli, H., Ali, A., Al Mannai, L., Aladab, A., AlSaadi, H., AlKarbi, K. A., & Al-Baghdadi, T. (2021). Psychological and Coping Strategies Related to Home Isolation and Social Distancing in Children and Adolescents During the COVID-19 Pandemic: Cross-sectional Study. *JMIR formative research*, *5*(4), e24760-e24760. https://doi.org/10.2196/24760

Zhao, Y., Guo, Y., Xiao, Y., Zhu, R., Sun, W., Huang, W., Liang, D., Tang, L., Zhang, F., Zhu, D., & Wu, J.-L. (2020). The Effects of Online Homeschooling on Children, Parents, and Teachers of Grades 1-9 During the COVID-19 Pandemic. *Medical science monitor : international medical journal of experimental and clinical research, 26*, e925591-e925591. https://doi.org/10.12659/MSM.925591

Zhu, S., Zhuang, Y., & Ip, P. (2021). Impacts on Children and Adolescents' Lifestyle, Social Support and Their Association with Negative Impacts of the COVID-19 Pandemic. *International journal of environmental research and public health, 18*(9). https://doi.org/10.3390/ijerph18094780
